# Cricothyrotomy in Acute Upper Gastrointestinal Bleed: A Difficult Airway Simulation Case for Anesthesiology Residents

**DOI:** 10.15766/mep_2374-8265.11378

**Published:** 2024-01-16

**Authors:** Corinna J. Yu, Frank Rigueiro, Kevin Backfish-White, Johnny Cartwright, Christopher Moore, Sally A. Mitchell, Tanna Boyer

**Affiliations:** 1 Assistant Professor, Department of Anesthesia, Indiana University School of Medicine; 2 Fourth-Year Medical Student, Indiana University School of Medicine; 3 Simulation Specialist, Department of Anesthesia, Indiana University School of Medicine; 4 Associate Professor, Department of Anesthesia, Indiana University School of Medicine

**Keywords:** Cricothyrotomy, Difficult Airway Algorithm, Acute Upper GI Bleed, Bleeding Airway, Emergency Intubation, Surgical Airway, Airway Simulation, Anesthesiology, Simulation, Editor's Choice

## Abstract

**Introduction:**

Patients with acute upper gastrointestinal bleeding may have challenging airways. This simulation teaches anesthesiology residents the skill of cricothyrotomy as a surgical last resort while managing acute bleeding in the airway.

**Methods:**

The simulation involved a 55-year-old patient with history of alcohol abuse admitted to the ICU with hematemesis and acute blood loss for esophagogastroduodenoscopy in the ICU setting. The mannequin had tubing in the posterior oropharynx connected to a pressurized bag of simulated blood hidden from view. While conversing, the patient began to cough and gag, and the bag of fluid was opened, filling the posterior oropharynx with blood, which prompted immediate intubation attempts, designed to fail no matter what the learners attempted. When residents requested a surgical airway, they were provided with a cricothyrotomy kit and a task trainer to perform the procedure. Residents were evaluated using a behavior checklist, debriefed, then asked to complete a postsimulation survey.

**Results:**

Fifty-eight anesthesiology residents completed the simulation and provided feedback via a 5-point Likert scale of agreement. Most residents quickly recognized the need for emergency intubation. Eighty-eight percent of participants strongly agreed that the simulation was a valuable learning experience, with 99% stating it increased their confidence and clinical decision-making in handling similar scenarios in the future.

**Discussion:**

This simulation provides a chance to practice valuable airway management skills that increase resident confidence in cricothyrotomy. Future work may examine if these skills and confidence levels are sustainable over time and if they are applied in future patient encounters.

## Educational Objectives

By the end of this activity, learners will be able to:

1. Formulate a plan for airway management in patients with acute upper gastrointestinal bleeding, including rapid sequence intubation and cricothyrotomy if necessary.
2. Acknowledge the resource limitations and planning necessary to safely provide anesthetics in out-of-OR locations.
3. Increase their confidence in the implementation of the difficult airway algorithm from the American Society of Anesthesiologists in a CICO (can't intubate, can't oxygenate) situation.
4. Practice performance of cricothyrotomy.

## Introduction

More than 400,000 patients are hospitalized in the United States every year for acute upper gastrointestinal bleeding (UGIB), with a mortality rate of 5%–10%.^1–3^ Acute UGIB has variceal and nonvariceal causes, which include peptic ulcer disease, erosive esophagitis, Mallory-Weiss tears, vascular malformations, and upper GI tract tumors and malignancies.^2^ The use of nonsteroidal anti-inflammatory drugs and antiplatelet and anticoagulant medications has also been associated with UGIB.^4^ Additional risk factors include age greater than 65, history of peptic ulcer disease, and male sex, with men having twofold higher risk of developing UGIB than women.^5^ There is also a twofold greater risk of rebleeding in patients with alcohol abuse, and excessive alcohol use is present in 20% of patients with UGIB.^6^

The standard of care for patients with UGIB is upper endoscopy within 24 hours of patient presentation.^2^ Anesthetic management often entails rapid sequence induction and endotracheal intubation, but ongoing bleeding may increase aspiration risk as well as the possibility of a difficult airway. In the event of failed intubation and ventilation attempts, the American Society of Anesthesiologists (ASA) difficult airway algorithm recommends emergency invasive airway access as a last resort.^7^ The advanced trauma life support manual recommends cricothyrotomy over tracheostomy because it is quicker, safer, easier to perform, and associated with less bleeding.^8^ Percutaneous dilatational tracheostomy is the most commonly used method of tracheostomy in the ICU, being used in nearly 75% of all recorded tracheostomies, making it a useful skill to teach to anesthesiology residents.^9^ Learners with specific tracheostomy training throughout residency showed increased confidence in handling such emergencies.^10^ Difficult airway management is often taught on a case-by-case basis, which leads to inconsistent training and residents lacking in confidence in performing needle cricothyrotomy.^11^ Although many anesthesiology residency programs have formal airway rotations, in one survey, about half of the responding American and Canadian anesthesiology residency program directors indicated that they did not have a formal rotation,^12^ which has spurred the development of yearlong airway management fellowships.^13,14^

Simulation is an established practice to rehearse skills for infrequent clinical scenarios. A clinical vignette guiding residents down the ASA difficult airway algorithm followed by a multimedia educational intervention demonstrated improvement in time to cricothyrotomy, using a marker to mimic a scalpel.^11^ Similarly, practicing cricothyrotomy on mannequins led to increased procedural success after four attempts.^15^ Anesthesiology residents who had performed four or more bedside percutaneous tracheostomies as first assist thought they could obtain an invasive airway in an emergency situation.^16^ Residents participating in a bedside tracheostomy service uniformly agreed that it was a positive experience in their training and increased their skill set.^16^

For anesthesiology training programs without a tracheostomy service, simulation is a useful tool to standardize training, increase compliance with guidelines, and improve performance of cricothyrotomy.^17^ One simulation workshop focused on bougie-assisted surgical cricothyrotomy in 16 anesthesiology residents, with a self-assessed improvement of skill from a mean of −1.1 to 2.9 on an 11-point Likert-type scale (−5 = *very bad,* 5 = *excellent*).^18^ The CICO (can't intubate, can't oxygenate) situation in a bleeding airway presents a unique challenge. Our simulation pairs a common UGIB presentation with an out-of-OR setting (ICU) and cricothyrotomy, targeting anesthesiology residents and their preoperative preparation, anesthetic induction plan in a bleeding airway, and management of intraoperative complications including appropriate use of the difficult airway algorithm leading to surgical airway access. This resource is novel due to the clinical presentation of a UGIB, which may not be recognized as a difficult airway by residents, and not a practice skill of cricothyrotomy alone.

## Methods

### Development

This simulation was conducted for PGY 4 (CA 3) anesthesiology residents (*n* = 58) in the simulation laboratory at the Indiana University School of Medicine as part of the simulation curriculum from 2019 to 2023. The simulation was not run in 2020 due to the COVID pandemic, and the postsimulation survey was completed by 3 out of the 4 years of participants. Residents were given the patient's history (Appendix A, Learner Preparation section), which included past medical history, medication list, and social history. Residents were also informed that the patient was scheduled to undergo urgent upper endoscopy for hematemesis and had a 20G peripheral IV in place. Prerequisite knowledge included the ASA difficult airway algorithm and basic understanding of the steps of a needle and/or knife cricothyrotomy, which were components of our CA 1 curriculum.

### Equipment/Environment

Successful enactment of this simulation case required access to a bleeding airway simulator or task trainer with capability to mimic difficult airway pathology (Appendix B has additional details on simulation materials). Additional cricothyrotomy trainers and simulated skin membrane made from platinum cure silicone rubber (Ecoflex 00-30 Parts A & B, Smooth-On, Inc.) and pourable urethane rubber compound (VytaFlex 30, Smooth-On, Inc.) provided further opportunities for practice that were repeatable in all simulation groups and did not involve cutting the neck skin of an expensive, higher-fidelity mannikin. Also necessary were an ICU ventilator and basic airway supplies for endotracheal intubation, including laryngoscopes with straight and curved blades, endotracheal tubes, laryngeal mask airways, stylets, bougies, syringes, functional Yankauer suction tip complete with tubing and canister, whistle-tip suction catheters, carbon dioxide detector, oral airway, nasal airway, bag valve mask, nasal cannula, and a cricothyrotomy kit. Vital sign monitors to display heart rate, blood pressure, pulse oximeter, EKG, respiratory rate, temperature, and end-tidal carbon dioxide were available (e.g., Laerdal LLeap virtual monitor system). Video laryngoscopes and fiberoptic bronchoscopes were optional. Sprays that smelled like vomit were used to mimic the smell of a UGIB. Labeled syringes with anesthesia induction and code drugs and additional syringes and needles were available. The environment imitated an ICU in an inpatient hospital setting. A 1-liter bag of crystalloid dyed red to simulate blood was connected to IV tubing threaded discreetly in the posterior oropharynx of the airway simulator. It was placed on a pressure bag for vigorous bleeding during the scenario when released and could be exchanged for full bags when empty to continue high-flow bleeding in the airway. We recommended that residents wear hospital OR attire, including surgical masks, scrubs, gloves, and appropriate shoe coverings.

### Personnel

In each simulation, learners were PGY 4 (CA 3) anesthesiology residents. An attending anesthesiologist facilitated the simulation and debriefed with good intentions at its conclusion. The facilitator played the voice of the mannequin patient to answer questions preoperatively pertaining to the history and physical exam. A technician for the bleeding airway simulator and LLeap virtual monitor was present to alter vital signs of the mannequin in real time as the residents progressed through the simulation with induction, intubation, and ventilation attempts. The facilitator guided the technician through apnea, hypoxia, tachycardia, hypotension, bradycardia, and realistic end-tidal carbon dioxide values with airway attempts and cardiac output. The facilitator and technician discussed the simulation in advance to ensure learning objectives would be met.

Although embedded participants are the gold standard and best practice in simulation, due to budgetary limitations we used anesthesiology residents not in the hot seat playing an anesthesiologist in the scenarios to fill the roles of surgeon, circulating nurse, and, if available, scrub tech. Once established as a practice, this has worked well in our simulation program for over 10 years. All anesthesiology residents were included in the prebrief, scenario, and debrief, but not all were in the hot seat, which rotated among the learners for the day over three scenarios. Given that all anesthesiology residents had participated, we included them all in our postsimulation surveys.

### Implementation

Prior to the start of the simulation, anesthesiology residents were assigned to the following health care team roles: anesthesiologist, anesthesiology resident, gastroenterologist, and scrub nurse. The embedded participants (gastroenterologist and scrub nurse) wore earpieces so that directions from the control room could be given to them. At the beginning of the case, the anesthesiology team entered the simulation room and was tasked with obtaining a patient history. The verbal responses of the patient were provided by faculty in the control room playing the mannequin's voice. The residents entered the room, introduced themselves, and were expected to obtain a history and investigate whether the patient was willing to receive blood products before they induced anesthesia with appropriate medications that would maintain blood pressure and heart rate while ensuring rapid sequence intubation.

During the history, the patient complained of not feeling well and provided information consistent with the History of Presenting Illness section of the case file (Appendix A). The patient then began to cough vigorously and to make gagging noises as an embedded participant was directed to initiate the filling of the oropharynx with simulated blood. As the oropharynx filled with blood, participants were expected to attempt to intubate to secure the lost, bloody airway. In the event of three failed intubation attempts, participants ought to then to perform cricothyrotomy. The mannequin was set up to encourage failed intubation attempts every time by insertion of a fixed hard cannula or PVC plastic below the level of the cords. Another option used in a different year included a mannequin that that had its vocal cords superglued shut (Appendix B describes variations on simulation materials). After either a successful intubation had been performed or a surgical airway had been achieved with cricothyrotomy, the simulation ended, and all participants took part in a debriefing. In addition to discussing the case and what had gone well or could have gone better, residents received feedback assessing their decision-making and actions taken throughout the encounter (Appendix C). At the end of the debriefing, each participant was given a link to complete the postsimulation feedback form (Appendix D), an anonymous survey developed to determine efficacy.

### Debriefing

The debriefing began with a general discussion of the participants’ perceptions of the simulation outcome. The facilitator asked how the participants felt about their performance and confidence during the simulation. Participants were encouraged to reflect and analyze their strengths and weaknesses in completing the simulation exercise. The rest of the debriefing was organized to resemble the flow of the simulation itself. Each educational objective was addressed and discussed, following the order established in the behavior checklist (Appendix C). For each behavior listed, the facilitator provided feedback on performance and addressed any knowledge gaps. Faculty discussed common management errors and challenges in the case. The debriefing concluded with the team reentering the simulation room for a brief discussion on various cricothyrotomy techniques and opportunities to practice on the simulation mannequin. Appendix E features a debriefing guide and links to guides on how to perform a cricothyrotomy.

### Assessment

The behavior checklist for airway management of acute UGIB (Appendix C) was developed by local expert consensus of simulation faculty to track and assess the decision-making of participants. Behaviors identified on the checklist were marked as either not done, partly done, or done, based on performance during the simulation. The checklist was created based on the most updated recommendations from the ASA difficult airway algorithm. During the debriefing session, the checklist was used to discuss important actions and interventions that had been overlooked. Feedback was also given on overall performance compared to best practice, as determined by the attending anesthesiologist leading the session.

## Results

Fifty-eight PGY 4 (CA 3) anesthesiology residents completed the simulation and evaluation form from 2019 to 2023. The simulation was run 15 times with four different facilitators. Each facilitator was an experienced anesthesiologist capable of directing the simulation technologist on how to adjust vital signs to match the actions of the residents in the simulation. Facilitators read the case scenario in advance, reviewed the ASA difficult airway algorithm, and familiarized themselves with the OR crash cart, airway equipment, simulator, cricothyrotomy task trainer, and pressurized bag of simulated blood connected to the posterior oropharynx. One of the challenges included cleaning up the simulated blood between repetitions of the simulation. If simulated odors were used, a fan dispersed the smell before the simulation was repeated.

Facilitators used the behavior checklist (Appendix C) to track and assess the decision-making of residents during the simulation. Commonly missed interventions in the preoperative evaluation of a patient with an acute UGIB included asking about most recent hematemesis and most recent alcohol intake and determining recent hemoglobin and blood availability. Most residents proceeded with appropriate induction medications for a rapid sequence intubation with cricoid pressure and called for help. Most residents changed operator, instrument, or positioning when intubation was difficult, but many made more than three attempts to intubate before opting for cricothyrotomy. Residents who used continuous suction with either the Yankauer or whistle-tip suction catheter were able to view the vocal cords in the simulator but unable to intubate since the vocal cords were obstructed below or superglued. Most residents did not place the patient in the head-down position during active hemorrhage. Many residents were too focused on the airway to treat hypotension and bradycardia in a timely fashion.

Thirty-seven of the 58 residents had at least one prior experience with managing the airway in an acute UGIB before this simulation (Figure). Quantitative analysis of the simulation was recorded on a 5-point Likert scale (1 = *Strongly disagree,* 2 = *Somewhat disagree,* 3 = *Neutral,* 4 = *Somewhat agree,* 5 = *Strongly agree*; Table). When residents were asked to rank their agreement with the statement “This simulation was a valuable learning experience,” 88% of participants reported strongly agreeing, with 98% in agreement. The experience gained by learners increased their confidence and clinical decision-making in handling similar scenarios in the future, with 90% of participants strongly agreeing and 99% in agreement. The overwhelming majority of participants scored the overall simulation experience exceptionally highly, with *strongly agree* responses averaging 90% of the time, total agreement for *strongly agree* and *somewhat agree* 99% of the time, and *neutral* responses yielding just 2% in the questionnaire. An area for improvement was “The anesthesia faculty made me feel comfortable and at ease during debriefing,” where one individual somewhat disagreed.

**Figure.**
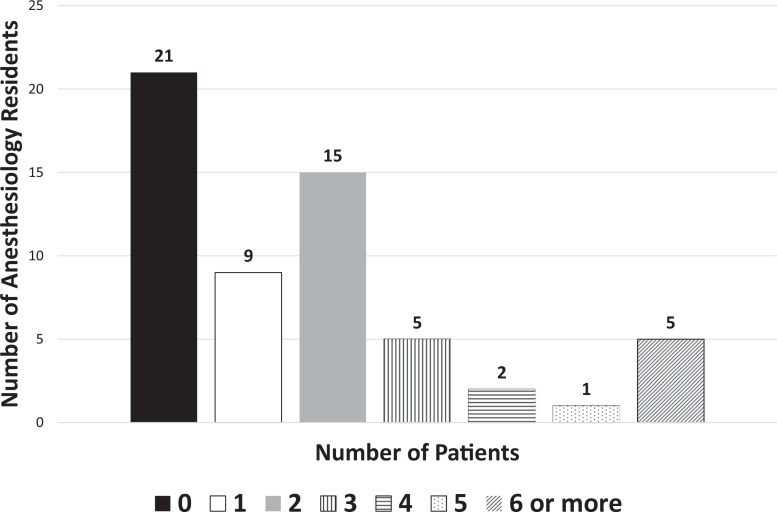
Prior experience of anesthesiology residents (*N* = 58) with managing the airway in an acute upper gastrointestinal bleed before the simulation.

**Table.**
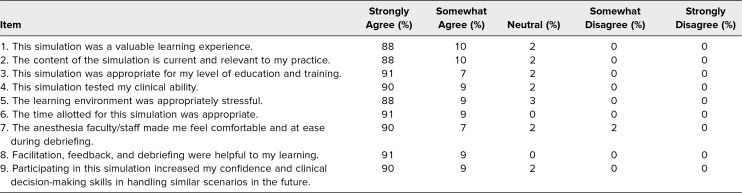
Postsimulation Survey Results (*N* = 58)

We also collected qualitative data, with an open-response section of the questionnaire asking participants to provide additional comments, observations, or feedback for improvement. Resident comments included “Good emphasis on creating as realistic an environment as possible,” “Very good simulation teaching surgical airway,” and “Realistic blood, great discussion post sim.” One resident commented on the odor used: “While at the time I didn't appreciate the smell, it really established the environment.” Another acknowledged their strengths and weaknesses, saying, “Good challenging scenario. Highly relevant and highlighted areas of strength and where I need to improve.”

## Discussion

Airway management is foundational to anesthesiology residency training, but variable clinical exposure to the difficult airway can make it difficult to assess this skill, especially in a stressful emergency. Formal instruction in invasive airway management training can improve procedural times for cricothyrotomy.^11^ Simulation provides a standardized approach to ensuring competency in the cricothyrotomy procedure. This simulation combined a challenging intubation with a UGIB to create a realistic difficult airway emergency forcing residents to proceed with a surgical airway. While the simulation led to an increase in resident confidence in the procedure, we did not follow up with the cohort to see if these skills prepared them for patient encounters afterwards and if the learning was sustainable.

In addition to the above limitation, other limitations included access to a simulator or task trainer with bleeding ability and a difficult airway. In the beginning, we created a difficult airway by obstructing the airway below the vocal cords with a large plastic piece in the trachea, making it completely impossible to intubate. Later, we superglued the vocal cords shut on a mannequin. Another option would be to inform the residents to assume that their intubation was esophageal and there was no end-tidal carbon dioxide while encouraging the embedded participants to push for a surgical airway. If the simulator did not have cricothyrotomy capabilities, the facilitator could pause the simulation, direct the residents to a cricothyrotomy trainer or model, and conclude the session with practicing cricothyrotomy, as was done in at least one of the years we ran the simulation.

Another limitation was including non-hot-seat learners in the postsimulation surveys. Given that not all surveyed residents played the anesthesiologist in the scenario and may have instead played a surgeon or circulating nurse, this could have affected their learning and responses. An additional limitation was the use of red saline versus simulated blood products, as the red saline was less viscous and lower fidelity than simulated blood products. This was done for two reasons. The first was the cost of simulated blood products, which would have included at least two bags per simulation over eight simulations per year; at the time of publication this would have been $300–$350 per year. The second reason was that, from a technical aspect, it was easier both to pump and to clean up the less viscous red saline than it would have been to clean up simulated blood products. If we were to use simulated blood products, we likely could only run one simulation per day, instead of our usual two, as the simulation specialists would need to take apart the mannequin head to clean it.

Simulation is the perfect venue to practice rare, life-threatening scenarios in a psychologically safe environment. Thus, cricothyrotomy has been simulated in other fields and is a well-published area.^11–18^ The most notable and recent cricothyrotomy simulation in *MedEdPORTAL* was published in 2021 by Asselin and colleagues; it focuses on adult cricothyrotomy skills in a 4-hour workshop format, taught in an interdisciplinary setting with both emergency medicine and anesthesiology residents and augmented for use during the COVID-19 pandemic.^18^ Our scenario is unique, specifically targeting the management of cricothyrotomy in a patient with an active UGIB in an outside-the-OR setting (intensive care unit). This oft-forgotten location of clinical practice among anesthesiology trainees brings a wealth of options for discussion and consideration when anesthesiologists are asked to practice outside the OR setting: What equipment is available, who and how do you call for help, what should you bring with you, and so on? Our scenario also emphasizes the challenges associated with bloody airway management and incorporates critical care principles, including hemodynamic monitoring, fluid resuscitation, coagulation management, and appropriate transfusion strategies. The scenario can also be set up easily to include intensivists-in-training and/or GI fellows, which could change the focus of the debriefing to include rapid decision-making, coordination among an interdisciplinary team, and proper communication strategies. A team of emergency medicine physicians has also trained paramedics on pediatric needle cricothyrotomy using simulation.^19^ Our scenario can be used for needle or knife cricothyrotomy training in adults, whereas that other one can only be used in pediatric patients, who at age 8 and under, are only eligible to receive a needle cricothyrotomy, thus leading to a different focus during the debriefing.

The simulation sessions in 2022–2023 included video recording, which allowed us to track duration of hypoxia. In future sessions with a set patient script and consistent timing for desaturation, tachycardia, hypotension, and bradycardia, we could track the time for intubation attempts before the request for surgical airway, as well as the time to complete the cricothyrotomy before and after the simulation training.

We believe it is worthwhile to include this simulation in the anesthesiology resident curriculum, in the PGY 4 (CA 3) year as it has a higher level of difficulty. The PGY 4 (CA 3) year setting ensures that all learners have the requisite knowledge of cricothyrotomy and statistically includes the highest probability that a learner in the simulation has attempted a cricothyrotomy in clinical practice, which elevates the discussion and impact upon the learners. This is one of the few simulations within our simulation curricula with a time- and simulation specialist-intensive technical setup. This time and technical challenge must be communicated to both simulation faculty and learners so that the simulation specialists has appropriate time and resources to plan and run the simulation as high fidelity as intended. The upside of this time and equipment setup is an extremely realistic simulation that makes the learners giddy as they genuinely enjoy the realistic blood spurting into the oropharynx of the mannequin.

In conclusion, our simulation case provides a valuable learning experience for anesthesiology residents and effective use of nonclinical educational time, as well as giving learners an opportunity to practice hands-on critical surgical airway skills in a psychologically safe environment. The case also includes a review of difficult airway management and where to find the evidence to guide difficult airway management in clinical practice, a valuable life skill for all practicing anesthesiologists. It may be useful for future competency-based residency curricula in airway management^20^ or for maintaining clinical competence in practicing anesthesiologists.^21^ This simulation could easily be used for critical care, emergency medicine, respiratory therapy, gastrointestinal, and surgical trainees with minimal modifications. It could also provide an opportunity to host interdisciplinary and/or interprofessional simulations between departments and hospital locations.

## Appendices


Simulation Case.docxSimulation Materials.docxBehavior Checklist.docxSimulation Feedback Form.docxDebriefing Guide.docx
*All appendices are peer reviewed as integral parts of the Original Publication.*

## References

[1] Aljarad Z, Mobayed BB. The mortality rate among patients with acute upper GI bleeding (with/without EGD) at Aleppo University Hospital: a retrospective study. Ann Med Surg (Lond). 2021;71:102958. 10.1016/j.amsu.2021.102958 34745601 PMC8551413

[2] Khamaysi I, Gralnek IM. Acute upper gastrointestinal bleeding (UGIB)—initial evaluation and management. Best Pract Res Clin Gastroenterol. 2013;27(5):633–638. 10.1016/j.bpg.2013.09.002 24160923

[3] Gralnek IM, Barkun AN, Bardou M. Management of acute bleeding from a peptic ulcer. N Engl J Med. 2008;359(9):928–937. 10.1056/NEJMra0706113 18753649

[4] Hreinsson JP, Kalaitzakis E, Gudmundsson S, Björnsson ES. Upper gastrointestinal bleeding: incidence, etiology and outcomes in a population-based setting. Scand J Gastroenterol. 2013;48(4):439–447. 10.3109/00365521.2012.763174 23356751 PMC3613943

[5] Hernández-Díaz S, Rodríguez LAG. Association between nonsteroidal anti-inflammatory drugs and upper gastrointestinal tract bleeding/perforation: an overview of epidemiologic studies published in the 1990s. Arch Intern Med. 2000;160(14):2093–2099. 10.1001/archinte.160.14.2093 10904451

[6] Kärkkäinen JM, Miilunpohja S, Rantanen T, et al. Alcohol abuse increases rebleeding risk and mortality in patients with non-variceal upper gastrointestinal bleeding. Dig Dis Sci. 2015;60(12):3707–3715. 10.1007/s10620-015-3806-6 26177705

[7] Apfelbaum JL, Hagberg CA, Connis RT, et al. 2022 American Society of Anesthesiologists practice guidelines for management of the difficult airway. Anesthesiology. 2022;136(1):31–81. 10.1097/ALN.0000000000004002 34762729

[8] Dillon JK, Christensen B, Fairbanks T, Jurkovich G, Moe KS. The emergent surgical airway: cricothyrotomy vs tracheotomy. Int J Oral Maxillofac Surg. 2013;42(2):204–208. 10.1016/j.ijom.2012.10.021 23265756

[9] Vargas M, Sutherasan Y, Antonelli M, et al. Tracheostomy procedures in the intensive care unit: an international survey. Crit Care. 2015;19:291. 10.1186/s13054-015-1013-7 26271742 PMC4536803

[10] Nizam AA, Ng SC, Kelleher M, Hayes N, Carton E. Knowledge, skills and experience managing tracheostomy emergencies: a survey of critical care medicine trainees. Ir Med J. 2016;109(9):471. 28125185

[11] Kahmke R, Crowson MG, Yalamuri S, Stolp BW, Puscas L, Woodard CR. Surgical airway education among anesthesia trainees: a cohort study. Glob Anesth Perioper Med. 2015;1(1):4–6. 10.15761/GAPM.1000102

[12] Pott LM, Randel GI, Straker T, Becker KD, Cooper RM. A survey of airway training among U.S. and Canadian anesthesiology residency programs. J Clin Anesth. 2011;23(1):15–26. 10.1016/j.jclinane.2010.06.009 21296243

[13] Koppel JN, Reed AP. Formal instruction in difficult airway management: a survey of anesthesiology residency programs. Anesthesiology. 1995;83(6):1343–1346. 10.1097/00000542-199512000-00025 8533927

[14] Straker T. Airway management: isn't that what anesthesiologists do? J Head Neck Anesth. 2019;3(1):e11. 10.1097/HN9.0000000000000011

[15] Wong DT, Prabhu AJ, Coloma M, Imasogie N, Chung FF. What is the minimum training required for successful cricothyroidotomy? A study in mannequins. Anesthesiology. 2003;98(2):349–353. 10.1097/00000542-200302000-00013 12552192

[16] Silverman RB, Quinn SM. Do anesthesia residents perceive a benefit from participating in bedside tracheostomies? J Educ Perioper Med. 2014;16(2):E068. 10.46374/volxvi-issue2-silverman 27175399 PMC4719549

[17] Hubert V, Duwat A, Deransy R, Mahjoub Y, Dupont H. Effect of simulation training on compliance with difficult airway management algorithms, technical ability, and skills retention for emergency cricothyrotomy. Anesthesiology. 2014;120(4):999–1008. 10.1097/ALN.0000000000000138 24434303

[18] Asselin M, Lafleur A, Labrecque P, Pellerin H, Tremblay MH, Chiniara G. Simulation of adult surgical cricothyrotomy for anesthesiology and emergency medicine residents: adapted for COVID-19. MedEdPORTAL. 2021;17:11134. 10.15766/mep_2374-8265.11134 33816795 PMC8015712

[19] Stopyra JP, Wright JL, Fitch MT, Mitchell MS. Pediatric needle cricothyrotomy: a case for simulation in prehospital medicine. MedEdPORTAL. 2017;13:10589. 10.15766/mep_2374-8265.10589 30800791 PMC6338176

[20] Galway UA, Straker T, Foley LJ, Aziz M, Woodworth G. Anesthesia residency training in airway management: a competency-based model curriculum. *A A Pract*. 2019;13(5):197–199. 10.1213/XAA.0000000000001046 31206383

[21] Bessman EL, Rasmussen LS, Konge L, et al.; Airway Management Education Study Group. Maintaining competence in airway management. Acta Anaesthesiol Scand. 2020;64(6):751–758. 10.1111/aas.13558 32034955

