# Supplementary material for: Cricothyrotomy in Acute Upper Gastrointestinal Bleed: A Difficult Airway Simulation Case for Anesthesiology Residents
Source: MedEdPORTAL. 2024 Jan 16;20:11378. doi: 10.15766/mep_2374-8265.11378 (PMC10789914; doi:10.15766/mep_2374-8265.11378)
Supplement: Supplementary file 1 — Simulation Case.docxSimulation Materials.docxBehavior Checklist.docxSimulation Feedback Form.docxDebriefing Guide.docx [file mep_2374-8265.11378-s001.zip › A. Simulation Case.docx]

| Appendix A: *MedEdPORTAL* Simulation Case  Cricothyrotomy in Acute Upper GI Bleed: A Difficult Airway Simulation Case for Anesthesiology Residents  Corinna Yu, MD, Frank Rigueiro, Kevin Backfish-White, MD, Johnny Cartwright, CHSOS-A, Chris Moore, BA, EMT-P, Sally A. Mitchell, EdD, MMSc, Tanna Boyer, DO | |
| --- | --- |
| PATIENT NAME: Jimmy Barnhardt  PATIENT AGE: 55-year-old male  CHIEF COMPLAINT: Hematemesis with anemia of acute blood loss, needs urgent upper endoscopy to evaluate  PHYSICAL SETTING: ICU with ventilator available | |
|  | |
| Brief narrative description of case | Jimmy Barnhardt is a 65-kg 55-year-old male who is scheduled to undergo urgent upper endoscopy for hematemesis. His past medical history includes hypertension on lisinopril and metoprolol daily, gastroesophageal reflux disease (no meds), and chronic alcohol abuse. He drinks 12 beers daily and has a working 20-gauge peripheral intravenous line in his right antecubital fossa.  Learners will be asked to interview the patient, obtain history and physical and consent as quickly as possible, during the interview the patient will cough up blood and lose consciousness, at which point no further history can be obtained and they will need to act quickly to rescue the patient and obtain an airway. The blood of the mannequin will quickly ooze blood. No matter what learners do, they will be unable to intubate the patient and will also be told they can no longer ventilate (can’t intubate can’t oxygenate or CICO), at which point they should attempt cricothyrotomy (with either needle or knife). The case ends after the cricothyrotomy is successful. |
| Primary Learning Objectives | 1. Ask appropriate questions during the preoperative Interview with a patient with an acute upper gastrointestinal bleed (UGIB). 2. Choose appropriate induction medications for a patient with an acute UGIB (rapid sequence induction with consideration for hypotension created by induction agents). 3. Practice airway management and intubation in a patient with an acute UGIB. 4. Discuss different intubation options available for difficult and bloody airways. 5. Discuss availability, or lack thereof, of equipment and help available in outside the operating room (OR) anesthetizing locations. 6. Use the ASA’s Difficult Airway Algorithm. 7. Practice needle or knife cricothyrotomy. |
| Critical Actions | 1. Introduce self to simulator. 2. Perform preoperative anesthetic evaluation. 3. Induce anesthesia appropriately given the clinical situation (rapid sequence induction with attempt to maintain blood pressure). 4. Attempt intubation in a bleeding airway. 5. Perform a needle cricothyroidotomy when clinically indicated. 6. Stabilize a bleeding hypotensive critical care patient in an out of OR setting. |
| Learner Preparation | It is assumed that the learners (PGY-4/CA-3) anesthesiology residents have been exposed to the ASA Difficult Airway Algorithm and cricothyrotomy as part of regularly scheduled lectures and difficult airway workshops in their residency curriculum. These topics are also tested on the American Board of Anesthesiology BASIC exam, which is taken at the end of PGY-2/CA-1 year.  Information below is given to the learners prior to the start of the simulation as part of the pre-brief. By this point in our simulation curriculum learners are very familiar with the fiction contract, psychological safety contract, and what the mannequin can do, but this is the point where these things would be emphasized for a different learner population.  Patient: Jimmy Barnhardt  55-year-old male  65-kg  No known drug allergies  Medications: lisinopril, metoprolol  Past Medical History: acute hematemesis, hypertension, gastroesophageal reflux disease (no medications), alcoholism  Past Surgical History: tonsillectomy and adenoidectomy as a child, no problems with general anesthesia  Family History: No anesthesia problems in family members  Social History: Drinks 12 beers daily  Scheduled to undergo urgent upper endoscopy for hematemesis.  He has a working 20 gauge peripheral intravenous line in his right antecubital fossa. |

| Initial Presentation | | | |
| --- | --- | --- | --- |
| Initial vital signs | Heart Rate: 101, Blood Pressure: 111/53, Oxygen Saturation: 98%, Respiratory Rate: 18, Temperature: 36.9 degrees Celsius | | |
| Overall Appearance | A pale, thin man, with bloody lips, stable, but becoming tachycardic and hypotensive. There is a suction canister in the room with 500-600 mL blood already in it. | | |
| Actors and roles in the room at case start | Resident Anesthesiologist  Attending Anesthesiologist  Gastroenterologist  Nurse (if enough residents present)  The resident and attending anesthesiologists are the primary learners in this scenario. Although the resident may initiate management first or defer to the attending, either the resident or attending may organically take the lead or take turns during the scenario as a team. If there are less residents, these roles can be consolidated into one role. The best educational simulation practice and gold standard is to have embedded participants play all non-anesthesiologist roles. If the program does not have budget and/or personnel to embed this number of participants, other learners can play the role of gastroenterologist and/or nurse using earpieces to guide their words and actions. If there are not enough embedded participants, the gastroenterologist and nurse can be consolidated into one role. | | |
| History of Presenting Illness | Patient volunteers he is feeling unwell and gives a good history. He does not volunteer how much blood he has vomited unless asked. He vomited some at home and seems to be working on filling up the canister here. “Can you get me a new one?” | | |
| Past Medical/Surgical History | Medications | Allergies | Family History |
| Hypertension on 2 medications  Gastroesophageal reflux disease (no medications)  Alcoholism  Tonsillectomy and adenoidectomy as a child, no problems with general anesthesia | Lisinopril  Metoprolol | No known drug allergies. | No problems with anesthesia. |
| Physical Examination | | | |
| General | Pale, thin, appears sickly | | |
| Head, Eyes, Ears, Nose, Throat | Blood around mouth, otherwise normal | | |
| Neck | Full range of motion, normal thickness | | |
| Lungs | Clear to auscultation bilaterally, no wheezes, rales, or rhonchi | | |
| Cardiovascular | Regular rate and rhythm, no murmurs, rubs, or gallops | | |
| Abdomen | Distended, nontender | | |
| Neurological | Alert and oriented to person, place, time | | |
| Skin | Thin, large veins visible | | |
| Genitourinary | Deferred | | |
| Psychiatric | Deferred, but appears normal | | |

| Instructor Notes - Changes and CASE Branch Points | | |
| --- | --- | --- |
| Intervention / Time point | Change in Case | Additional Information |
| All residents are given information about the patient’s medical history and the need for urgent esophagogastroduodenoscopy (EGD). They are given the opportunity to ask additional questions as a group before assuming their assigned roles. |  | If a resident asks for the starting hemoglobin: “The hemoglobin is 8 g/dL.”  If a resident asks for a second peripheral intravenous line: “You have an 18-gauge peripheral intravenous line in the left forearm.”  If a resident asks if they need consent: “This is an emergency procedure and two-physician consent will suffice.” |
| The residents move to the operating area and assume their assigned roles. The resident and attending anesthesiologist prepare medications and airway equipment for a rapid sequence intubation. They may ask for difficult airway equipment or blood to be available. | The resident or attending anesthesiologist fail to choose appropriate induction drugs or select a laryngeal mask airway instead of an endotracheal tube. | The nurse asks, “Do you need any other medications?” “Do you have everything you need?” “Would you like any additional equipment?” The gastroenterologist or nurse can ask, “Do you want blood available?” |
| The resident anesthesiologist interviews the patient first, with the attending anesthesiologist following with any additional questions afterwards. The simulation specialist answers questions for the mannequin. | The resident anesthesiologist fails to ask about nothing by mouth (NPO) status and last vomitus. | The patient complains, “I’ve been sick to my stomach and throwing up every 5 minutes!” |
| The resident and attending anesthesiologist begin to interview the patient, but the interview is cut short after 2-3 minutes because the patient starts vigorously coughing then begins to vomit and loses responsiveness. | The resident and attending anesthesiologist call for help and urgently proceed to secure the airway. | If the resident or attending anesthesiologist continues to ask questions during the coughing fit, the simulator can shake their head if possible and does not respond to additional questions. The nurse asks, “Can I call someone to help?” They make a phone call, hang up, and respond, “They’re all in cases right now.” The gastroenterologist asks, “Do we need an airway?” |
| After successful induction of anesthesia, the patient falls asleep, and the oropharynx begins to fill with blood. | The resident and attending anesthesiologist try to suction or intubate the patient. Suction is initially turned off so the team must troubleshoot fixing the suction. | Vitals remain stable if induction dosing is appropriate. If too large a dose of medication is given, the patient becomes hypotensive and tachycardic with heart rate in the 120s beats per minute and BP 80s/40s mmHg. If the patient was not preoxygenated, pulse oximetry oxygen saturation drops to 60% over 30 seconds. The nurse asks, “Is your suction turned on?” while the gastroenterologist states, “Your suction isn’t on!” |
| The resident and attending anesthesiologist request additional airway materials. |  | Video laryngoscopes, fiberoptic bronchoscopes, bougies, laryngeal mask airways, stylets, are all available. |
| The resident or attending anesthesiologist requests cricoid pressure. |  | The nurse applies cricoid pressure. |
| The resident or attending anesthesiologist requests Trendelenburg position. |  | The nurse changes the bed position. |
| The resident or attending anesthesiologist asks for intravenous fluids. |  | The simulation specialist responds, “Intravenous fluids are wide open.” |
| The resident or attending anesthesiologist asks for blood products. |  | The nurse responds, “2 units of packed red blood cells are on their way.” |
| The resident or attending anesthesiologist gives blood products and/or pressors. | The patient becomes less hypotensive. | The blood pressure stabilizes to baseline and heart rate returns to 100 bpm with appropriate pressor doses. |
| The resident or attending anesthesiologist persists in attempting to intubate with the same equipment. | The nurse and gastroenterologist offer to help. Pulse oximetry oxygen saturation decreases slowly over 3-5 minutes to low 20%. | The nurse asks, “Can I get you anything else?” “Would you like cricoid pressure?” while the gastroenterologist asks, “Do you want me to try?” The resident or attending anesthesiologist should ask for a cricothyrotomy kit and begin to perform the procedure. |
| The resident or attending anesthesiologist requests a pulse check. |  | The gastroenterologist responds, “It’s thready, but present.” |
| The resident or attending anesthesiologist requests additional help from other anesthesiologists. |  | The nurse picks up a phone then hangs up, saying, “They’re all in a trauma right now.” |
| The resident or attending anesthesiologist requests additional help from a surgeon for a surgical airway. |  | The nurse answers, “The surgeon is on their way but it will be 30 minutes.” |
| The resident or attending anesthesiologist intubates the esophagus. | Simulated blood pours out of the endotracheal tube. | The gastroenterologist asks, “Should we apply suction to that?” |
| The resident or attending anesthesiologist does not initiate cricothyrotomy in a timely fashion. | Pulse oximeter waveform disappears, end-tidal carbon dioxide begins to drop to 15 mmHg, heart rate initially increases to 150 bpm with hypotension to 60/30 mmHg. Ultimately patient becomes bradycardic to 30 bpm and blood pressure cuff cycles without a reading. The nurse begins to open the cricothyrotomy kit. | The nurse asks, “Should we do a cricothyrotomy?” The gastroenterologist asks, “Do we need to begin advanced cardiac life support (ACLS)?” |
| The resident or attending anesthesiologist attempts placement of a laryngeal mask airway. | No end-tidal carbon dioxide on the monitor. |  |
| The resident or attending anesthesiologist performs a cricothyrotomy (needle or knife). | End-tidal carbon dioxide returns, breath sounds auscultated bilaterally. Pulse oximetry begins to increase, heart rate and blood pressure may improve. |  |
| The resident or attending anesthesiologist requests to call a code blue. | The nurse or simulation specialist brings the code cart into the room. Proceed with a mock code following ACLS including defibrillation with return of spontaneous circulation after cricothyrotomy is placed. | Do not allow the patient to die as the goal of this scenario is not a death discussion. |

Ideal Scenario Flow

The residents enter the room and introduce themselves to the patient. They explain that he is high risk for having an anesthetic today but that it is a risk he must take to control the bleeding. They should ask if he is willing to receive blood products. They will prepare their airway equipment and induce anesthesia appropriately given the clinical situation (rapid sequence induction), choosing drugs that will keep vital signs stable. During preoperative evaluation, induction, and the first intubation attempt, blood fills the oropharynx. Residents will be unable to intubate due to sim specialist changes to the mannequin. If they are unsuccessful 3 times after changing instruments and operators, they should perform a cricothyrotomy. The scenario ends after a successful cricothyrotomy.

Anticipated Management Mistakes

1. Inadequate preparation: Residents fail to prepare supplies, set up, and check for backup airway equipment (intubating blades, endotracheal tubes, bougie, videolaryngoscope, fiberoptic bronchoscope, suction). The suction may not be turned on, may stop working after the first canister is filled, and could require troubleshooting.
2. Failure to respond to an emergent situation: When the patient begins to vomit and becomes unresponsive, residents may struggle to switch from preoperative history-taking to emergent management of the patient in a timely fashion.
3. Inappropriate selection of medications and dosages: Residents may choose induction agents and dosages that may cause greater hypotension and tachycardia in a patient who is bleeding. They may neglect to perform a rapid sequence induction with appropriate paralytic leading to the patient aspirating.
4. Failure to manage vital signs: Residents may focus exclusively on airway management and forget to manage hypotension, tachycardia. They may delay the initiation of compressions if necessary for ACLS.
5. Delay in proceeding with the emergency pathway: Residents may attempt too many times to intubate without changing instrument or operator and be unaware of the passage of time, spending too much time with the patient decompensating before resorting to a surgical airway. They may persist in using videolaryngoscopes or fiberoptic bronchoscopes in a futile fashion with a bloody airway.
