# Supplementary material for: Cricothyrotomy in Acute Upper Gastrointestinal Bleed: A Difficult Airway Simulation Case for Anesthesiology Residents
Source: MedEdPORTAL. 2024 Jan 16;20:11378. doi: 10.15766/mep_2374-8265.11378 (PMC10789914; doi:10.15766/mep_2374-8265.11378)
Supplement: Supplementary file 1 — Simulation Case.docxSimulation Materials.docxBehavior Checklist.docxSimulation Feedback Form.docxDebriefing Guide.docx [file mep_2374-8265.11378-s001.zip › B. Simulation Materials.docx]

**Appendix B. Simulation Materials**

Initially, we used a head and trunk mannequin that was no longer functional and out of warranty. We used a fixed hard cannula through the anterior neck to create a physical obstruction behind the vocal cords so that endotracheal tubes could not be passed. This later changed to a PVC plastic barrier behind the vocal cords. We hid tubing behind the soft palate of the mannequin and an embedded participant opened the pressurized bag of red fluid during the simulation to pour blood into the airway. When learners identified the need for cricothyrotomy, the simulation was ended and cricothyrotomy task trainers were brought out for the learners to practice the skill using expired cricothyrotomy kits. The task trainers included reusable skin over replaceable tape as the airway barrier.


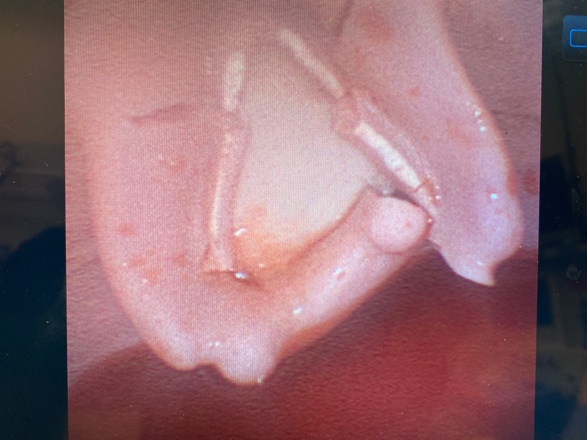

© 2023 Corinna Yu

*Image 1. PVC plastic behind vocal cords to create physical barrier to intubation and “difficult intubation.”*

In subsequent iterations of the simulation, the head and neck mannequin was placed above a high fidelity full body mannequin to make the simulation more realistic. The vocal cords were superglued shut so they were impenetrable.


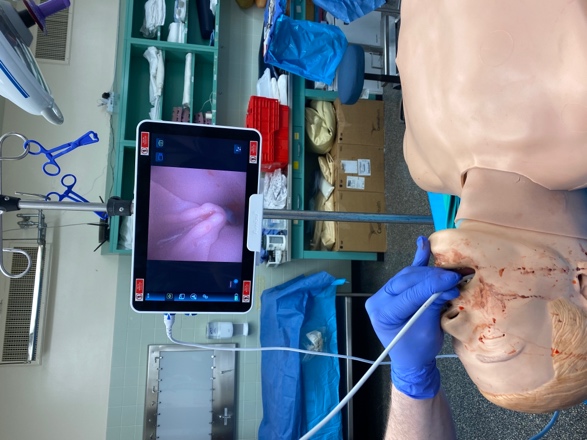

© 2023 Corinna Yu

*Image 2. Flexible bronchoscope image of superglued vocal cords.*

The learners performed the cricothyrotomy directly on the mannequin. The larynx was sculpted out of clay to make a silicone mold and then filled with urethane rubber to make the larynx. Skin overlay was made from silicone to place on top.


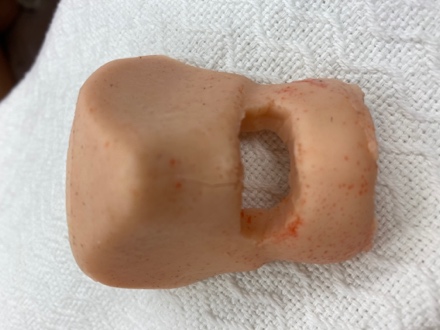

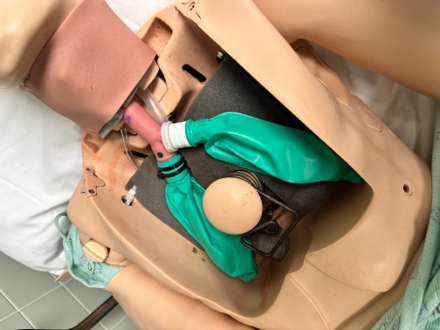

© 2023 Corinna Yu

*Images 3 & 4. Left: Urethane rubber larynx made from a hand-sculpted silicone mold. Right: Silicone skin on mannequin.*

In addition, instead of requiring someone to manually release the pressurized blood to simulate the GI bleed, a submersible high flow aquarium water pump was used in a large 5-gallon bucket. The bucket was filled with 2/3 distilled water and a container of VATA simulated blood. Tubing was threaded through the esophagus using ¾ inch tubing and a quick connect. The GI bleed was simulated using a remote control for the water pump, which was hidden underneath the surgical drapes under the foot of the bed. Simulated blood was mixed with liquid solidifier and sprayed with BARFume Puke Spray to simulate the smell and appearance of gastric contents from a GI bleed.


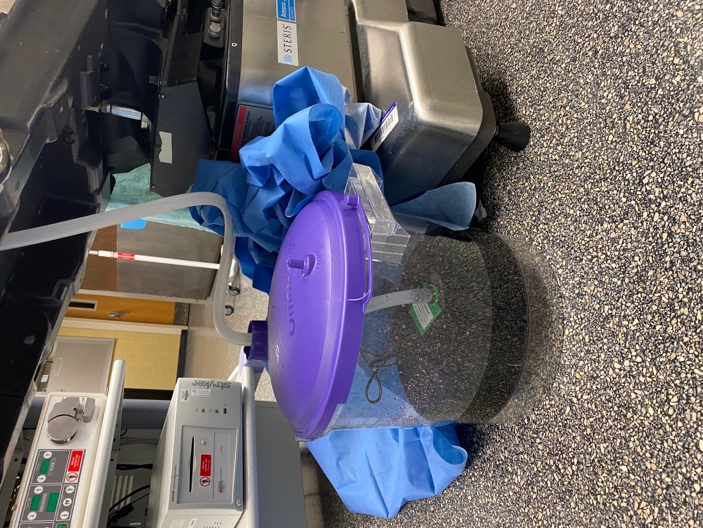

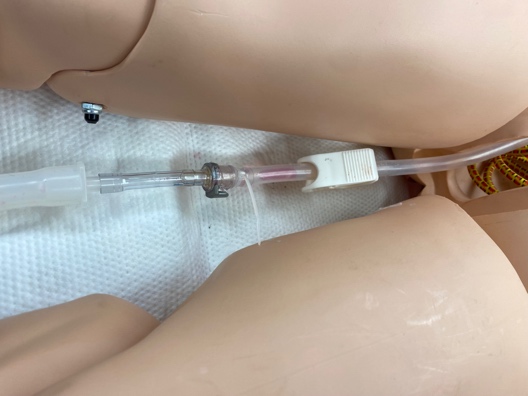

© 2023 Corinna Yu

*Images 5 & 6. Left: Bucket with aquarium pump and simulated blood. Right: Quick connect tubing.*


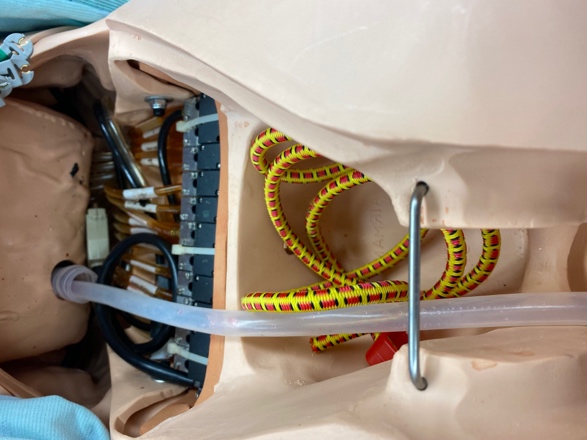

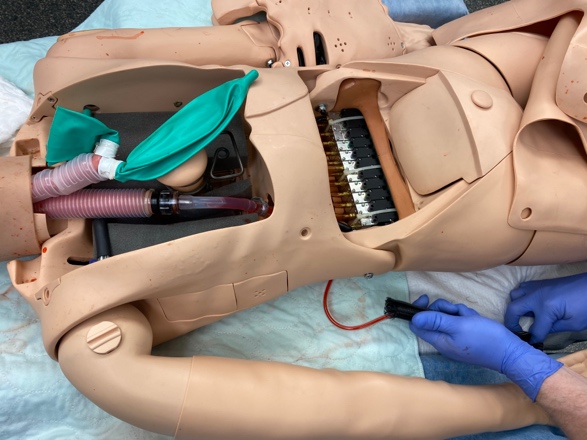

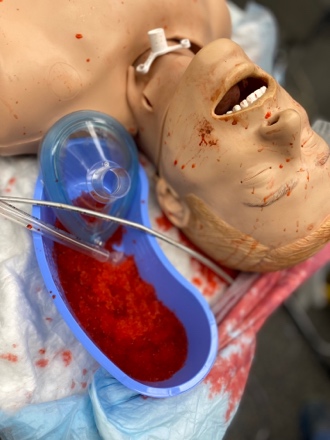

© 2023 Corinna Yu

*Images 7 & 8. Left: Tubing traversing into chest cavity. Center: Tubing into esophagus. Right: Simulated blood with liquid solidifier and BARFume Puke Spray to simulate gastric contents from a GI bleed.*


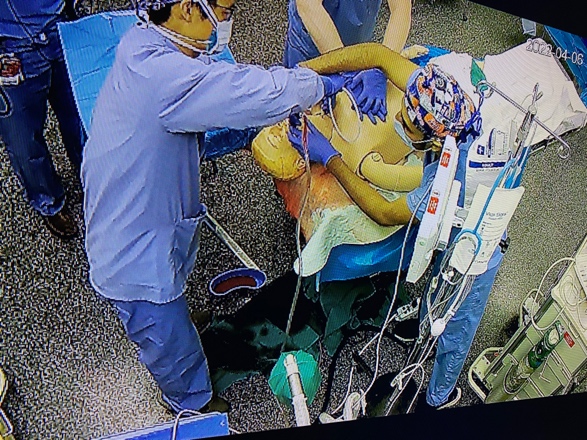

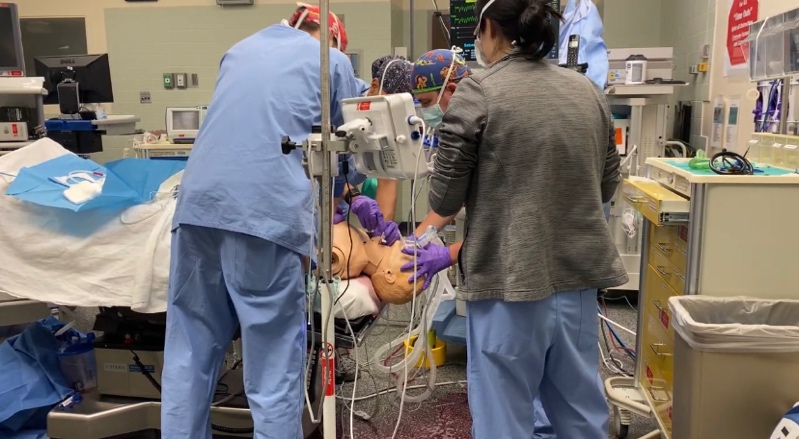

© 2023 Corinna Yu

*Images 9 & 10. Left: Anesthesiology residents working on suctioning simulated blood from the oropharynx during intubation attempts while beginning CPR. Right: Anesthesiology residents placing a cricothyrotomy while performing CPR during a code.*
