# Supplementary material for: Cricothyrotomy in Acute Upper Gastrointestinal Bleed: A Difficult Airway Simulation Case for Anesthesiology Residents
Source: MedEdPORTAL. 2024 Jan 16;20:11378. doi: 10.15766/mep_2374-8265.11378 (PMC10789914; doi:10.15766/mep_2374-8265.11378)
Supplement: Supplementary file 1 — Simulation Case.docxSimulation Materials.docxBehavior Checklist.docxSimulation Feedback Form.docxDebriefing Guide.docx [file mep_2374-8265.11378-s001.zip › C. Behavior Checklist.docx]

**Appendix C: Behavior Checklist for Airway Management of an**

**Acute Upper Gastrointestinal Bleed Simulation**

|  | **Expected Actions** | **NOT DONE** | **PARTLY DONE** | **DONE** |
| --- | --- | --- | --- | --- |
| **Preoperative Evaluation** | Appropriate introduction |  |  |  |
|  | Asks about NPO status |  |  |  |
|  | Asks about most recent hematemesis |  |  |  |
|  | Asks about most recent alcoholic intake |  |  |  |
|  | Asks about recent labwork and starting Hb |  |  |  |
|  | Asks about blood availability for case |  |  |  |
|  | | | | |
| **Intraoperative Management** | Chooses appropriate induction medications and dosages |  |  |  |
|  | Chooses appropriate paralytic for rapid sequence intubation (if applicable) |  |  |  |
|  | Calls for help when patient status becomes critical |  |  |  |
|  | Communicates situation to OR staff |  |  |  |
|  | Changes operator, instrument, or positioning with difficult intubation |  |  |  |
|  | Suctions blood |  |  |  |
|  | Places patient in head down position |  |  |  |
|  | Asks for cricothyroidotomy kit |  |  |  |
|  | Asks for surgeon for potential surgical airway |  |  |  |
|  | Treats blood pressure appropriately with blood or pressors |  |  |  |
