# Supplementary material for: Cricothyrotomy in Acute Upper Gastrointestinal Bleed: A Difficult Airway Simulation Case for Anesthesiology Residents
Source: MedEdPORTAL. 2024 Jan 16;20:11378. doi: 10.15766/mep_2374-8265.11378 (PMC10789914; doi:10.15766/mep_2374-8265.11378)
Supplement: Supplementary file 1 — Simulation Case.docxSimulation Materials.docxBehavior Checklist.docxSimulation Feedback Form.docxDebriefing Guide.docx [file mep_2374-8265.11378-s001.zip › D. Simulation Feedback Form.docx]

**Appendix D: SIMULATION FEEDBACK FORM**

**TITLE:** Airway Management in an Acute Upper Gastrointestinal Bleed: An Anesthesia Simulation Case for Anesthesiology Residents

**Date:**

**Facilitator(s):**

**Learner’s Level of Training** (PGY-1, PGY-2, etc): PGY-1 / PGY-2 / PGY-3 / PGY-4 / PGY-5

**Have you ever taken care of a patient similar to this scenario before?** Yes / No

|  | **Strongly Disagree** | **Disagree** | **Neutral** | **Agree** | **Strongly Agree** |
| --- | --- | --- | --- | --- | --- |
| This simulation was a valuable learning experience. |  |  |  |  |  |
| The content of the simulation is current and relevant to my practice. |  |  |  |  |  |
| This simulation was appropriate for my level of education and training. |  |  |  |  |  |
| This simulation tested my clinical ability. |  |  |  |  |  |
| The learning environment was appropriately stressful. |  |  |  |  |  |
| The time allotted for this simulation was appropriate. |  |  |  |  |  |
| The anesthesia faculty/staff made me feel comfortable and at ease during debriefing. |  |  |  |  |  |
| Facilitation, feedback, and debriefing were helpful to my learning. |  |  |  |  |  |
| Participating in this simulation increased my confidence and clinical decision-making skills in handling similar scenarios in the future. |  |  |  |  |  |

**Additional Comments/Observations/Feedback for Improvement:**
