# Supplementary material for: Cricothyrotomy in Acute Upper Gastrointestinal Bleed: A Difficult Airway Simulation Case for Anesthesiology Residents
Source: MedEdPORTAL. 2024 Jan 16;20:11378. doi: 10.15766/mep_2374-8265.11378 (PMC10789914; doi:10.15766/mep_2374-8265.11378)
Supplement: Supplementary file 1 — Simulation Case.docxSimulation Materials.docxBehavior Checklist.docxSimulation Feedback Form.docxDebriefing Guide.docx [file mep_2374-8265.11378-s001.zip › E. Debriefing Guide.docx]

**Appendix E. Debriefing Materials**

The Promoting Excellence And Reflective Learning in Simulation (PEARLS) Healthcare Debriefing Tool^1^ is a useful framework to debrief the session.

1. **Setting the Scene:** Transition the learners from the stressful simulation environment and sit down in a neutral location. Explain the goal to debrief for the purpose of improving teamwork and patient care. Ensure psychological safety by stressing that everyone is intelligent and focused on improvement.

*“Let’s spend some time debriefing the scenario. Everyone here is talented and we’re here to learn so we can take better care of our patients.”*

1. **Reactions:** Solicit initial reactions and emotions.

*“What did you think? How did that feel?”*

1. **Description:** Develop a shared mental model of the course of events.

*“Can someone summarize what happened in the simulation case? What went well? What could be improved? What was the differential diagnosis and treatment plan?”*

1. **Analysis:**
   1. **Learner Self-Assessment:** Focus on the performance domains of situational awareness, decision making, and communication.

*“When did you become aware of the potential for a difficult airway? What was your thought process when the patient began vomiting blood? How did you decide to manage the airway and why? How could communication of the critical situation be improved?”*

- 1. **Focused Facilitation:** Focus on resource utilization. Discuss the airway management techniques used and review the ASA Difficult Airway Algorithm^2^ as a comparison.

*“I saw you use [insert airway management tools] to try to intubate. Are there any other airway tools you could have used? What did you think about your choice in medications for rapid sequence induction? Which tools did you wish you had available to you sooner and why? Did you change operators and call for help? How did you feel about the passage of time?”*

- 1. **Provide Information:** Focus on technical skills.

*“How did you feel about the technical performance of the cricothyrotomy? Does anyone have any questions about the steps of the procedure?”*

Review the steps using the following resources:

- - 1. [Merck Manual: How To Do A Percutaneous Cricothyrotomy](https://www.merckmanuals.com/professional/critical-care-medicine/how-to-do-other-airway-procedures/how-to-do-a-percutaneous-cricothyrotomy)^3^
    2. [Stat Pearls: Cricothyrotomy](https://www.merckmanuals.com/professional/critical-care-medicine/how-to-do-other-airway-procedures/how-to-do-a-percutaneous-cricothyrotomy)^4^

1. **Application/Summary:** Identify take-aways from the simulation session.

*“As you go into clinical practice, what will you remember from today’s session?”* Allow for silence if necessary to allow the learner to process their thoughts.

*“The key learning points for the case were to identify that a patient with an acute upper gastrointestinal bleed can be a difficult airway, be prepared with your airway management plan including backup plans, use the ASA Difficult Airway Algorithm effectively, and practice performing a cricothyrotomy.”*

There are many effective and easily available manuals for performing cricothyrotomy that you can use for faculty review and to share with learners. Our favorites are referenced below. Given generational preferences of learners, videos may actually be preferred by today’s resident learners over step-by-step guides or references to article publications.

1. Emergency Three Step Knife Cricothyrotomy^5^ - <https://academic.oup.com/milmed/article/172/12/1228/4627002>
2. Needle Cricothyrotomy^6^ – Seldinger technique – likely available through your institution’s library <https://accessmedicine.mhmedical.com/content.aspx?bookid=2969&sectionid=250463597>
3. Percutaneous (needle) Cricothyrotomy^7^ – Seldinger technique – possibly available through your institution’s library - <https://app.jove.com/v/10239/percutaneous-cricothyrotomy>
4. Needle Cricothyrotomy video^8^ – free on YouTube - [Needle Cricothyroidotomy](https://www.youtube.com/watch?v=fNRDWN2OdpY)
5. Seldinger Technique Cricothyrotomy video^9^ – free on YouTube - Cricothyroidotomy: [Seldinger technique](https://www.youtube.com/watch?v=GewdLGcnaaY)

**References:**

1. Bajaj K, Meguerdichian M, Thoma B, et al. The PEARLS Healthcare Debriefing Tool. *Academic Medicine*. 2018;93(2):336. <https://doi.org/10.1097/ACM.0000000000002035>
2. Apfelbaum JL, Hagberg CA, Connis RT, et al. 2022 American Society of Anesthesiologists Practice Guidelines for Management of the Difficult Airway. *Anesthesiology*. 2022;136(1):31-81. <https://doi.org/10.1097/ALN.0000000000004002>
3. Chappell B. (2023, Feb). *How To Do A Percutaneous Cricothyrotomy*. Merck Manual. <https://www.merckmanuals.com/professional/critical-care-medicine/how-to-do-other-airway-procedures/how-to-do-a-percutaneous-cricothyrotomy>.
4. McKenna P, Desai NM, Tariq A, et al. *Cricothyrotomy*. StatPearls [Internet]. <https://www.ncbi.nlm.nih.gov/books/NBK537350/?report=reader>.
5. Allan MacIntyre, DO and others, Three-Step Emergency Cricothyroidotomy, *Military Medicine*, Volume 172, Issue 12, December 2007, Pages 1228–1230, <https://doi.org/10.7205/MILMED.172.12.1228>
6. White SJ, High K, Stack LB, Levitan RM. Cricothyrotomy (Seldinger Technique). In: Knoop KJ, Stack LB, Storrow AB, Thurman R. eds. *The Atlas of Emergency Medicine, 5e*. McGraw Hill; 2021. Accessed July 07, 2023. <https://accessmedicine.mhmedical.com/content.aspx?bookid=2969&sectionid=250463597>
7. JoVE Science Education Database. Emergency Medicine and Critical Care. Percutaneous Cricothyrotomy. JoVE, Cambridge, MA, (2023).
8. EMCape Town. Needle Cricothyrotomy. YouTube. Feb 1, 2013. <https://www.youtube.com/watch?v=fNRDWN2OdpY>. Accessed July 7, 2023.
9. National Tracheostomy Safety Project. Cricothyroidotomy: Seldinger Technique. YouTube. November 18, 2012. <https://www.youtube.com/watch?v=GewdLGcnaaY.> Accessed July 7, 2023.
